# DNA Sensor for the Detection of *Brucella* spp. Based on Magnetic Nanoparticle Markers

**DOI:** 10.3390/ijms242417272

**Published:** 2023-12-08

**Authors:** Abdalhalim Abuawad, Yaqoub Ashhab, Andreas Offenhäusser, Hans-Joachim Krause

**Affiliations:** 1 Institute of Biological Information Processing: Bioelectronics (IBI-3), Forschungszentrum Jülich, 52428 Jülich, Germany; a.abuawad@fz-juelich.de (A.A.);; 2 Faculty of Mathematics, Computer Science and Natural Sciences, Rheinisch-Westfälische Technische Hochschule Aachen University, 52062 Aachen, Germany; 3 Palestine–Korea Biotechnology Center, Palestine Polytechnic University, Hebron P720, Palestine

**Keywords:** magnetic nanoparticles, frequency mixing magnetic detection, DNA biosensor, brucellosis, point of care testing, infectious diseases

## Abstract

Due to the limitations of conventional *Brucella* detection methods, including safety concerns, long incubation times, and limited specificity, the development of a rapid, selective, and accurate technique for the early detection of *Brucella* in livestock animals is crucial to prevent the spread of the associated disease. In the present study, we introduce a magnetic nanoparticle marker-based biosensor using frequency mixing magnetic detection for point-of-care testing and quantification of *Brucella* DNA. Superparamagnetic nanoparticles were used as magnetically measured markers to selectively detect the target DNA hybridized with its complementary capture probes immobilized on a porous polyethylene filter. Experimental conditions like density and length of the probes, hybridization time and temperature, and magnetic binding specificity, sensitivity, and detection limit were investigated and optimized. Our sensor demonstrated a relatively fast detection time of approximately 10 min, with a detection limit of 55 copies (0.09 fM) when tested using DNA amplified from *Brucella* genetic material. In addition, the detection specificity was examined using gDNA from *Brucella* and other zoonotic bacteria that may coexist in the same niche, confirming the method’s selectivity for *Brucella* DNA. Our proposed biosensor has the potential to be used for the early detection of *Brucella* bacteria in the field and can contribute to disease control measures.

## 1. Introduction

Zoonotic diseases are globally threatening both public health and economic sectors. The threat arises from the fact that these infectious diseases have the potential to cause new widespread outbreaks and become either epidemic or pandemic [1]. Brucellosis is one of the most common global zoonotic diseases that is endemic in several developing countries in the Middle East, Asia, and Africa [2]. Numerous studies showed an increased incidence rate of brucellosis in different geographical areas, highlighting the high risk of a reemergence of this disease [3,4]. Preventing the spread of the disease through a rigorous surveillance and control programs is essential to reduce the infection rate and minimize the economic and public health burden of this serious disease. The accurate and fast detection of infected animal samples in the field has been a major hurdle in applying efficient surveillance programs in endemic countries. Therefore, developing point-of-care testing (POCT) methods that can detect *Brucella* in the field is greatly needed to fight this zoonotic disease. POCT offers several benefits in terms of portability, rapid diagnosis, and low cost, which is critical for on-site detection [5].

Due to the limitations related to the long incubation time and safety issues of the method considered the gold standard, i.e., blood culture, serological assays like the rose bengal test (RBT) and the standard agglutination test (SAT) were used as rapid and safe tests for the detection of *Brucella* antibodies [6]. Despite their simplicity, these serological methods suffer from low specificity due to cross-reactivity with other bacterial organisms, as well as from low sensitivity at the early stage of infection [7,8]. Nucleic acid amplification tests (NAATs) like polymerase chain reaction (PCR) and its variants were proven to be more sensitive, specific, and safe than other conventional methods, which makes them a reliable alternative [9,10]. However, the requirements of sophisticated labs with different operational areas and stationary expensive equipment limit their practical use in the on-site testing of *Brucella*. Thus, the development of a suitable assay for the in-field testing of *Brucella* DNA is crucial to ensure a highly accurate and specific screening method.

The chemical stability, biocompatibility, and large surface area of nanoparticles make them highly attractive materials for the on-site detection of various target analytes [11]. Among nanoparticles, magnetic nanoparticles (MNPs) have been widely used in several nucleic acid assays, such as DNA magnetic extraction, target enrichment, and DNA detection based on MNPs functioning as sensing elements [12]. MNPs have advantages over other nanoparticles in biosensing assays, as they are highly responsive and controllable when a magnetic field is applied, leading to highly selective and rapid target detection [13]. The first advantage is that they have a high saturation magnetization, which leads to a strong response, allowing fast detection. Moreover, their specific magnetic response allows for accurate and efficient detection. Furthermore, they exhibit higher stability and minimal low background noise compared with other optical nanoparticles [14]. These advantages make them good candidates for reliable field detection of DNA.

The detection of MNPs can be achieved by using different methods such as susceptometry, relaxometry, and frequency mixing magnetic detection (FMMD) [15,16]. Among these techniques, FMMD, with its portable magnetic reader, has been widely used as a selective technique for detecting and quantifying superparamagnetic nanoparticles (MNPs). The technique utilizes the mixing of magnetic fields at different frequencies at the nonlinear magnetization of MNPs, generating a distinct magnetic response signal at the sum and difference frequencies that enables the specific detection of superparamagnetic nanoparticles. In the FMMD technique, the MNPs serve as markers to detect a wide range of analytes such as toxins [17], antigens [18], antibodies [19], and viruses [20]. In addition, multiplex detection of different particles was demonstrated, showing the potential of this technique to detect multiple analytes in a single assay [21,22].

In our study, we developed a magnetic nanoparticle-based biosensor for detecting *Brucella* DNA using our novel frequency mixing magnetic detection as a readout technique. Superparamagnetic nanoparticles were used as markers for sensing and quantifying the sequence-specific hybridization event between a capture probe and its complementary target DNA on polyethylene (PE) filters. This novel biosensor offers the advantage of portable on-site detection of amplified *Brucella* DNA at resource-limited locations for point-of-care testing.

## 2. Results and Discussion

### 2.1. Principle of the DNA Magnetic Assay

**Scheme 1:**
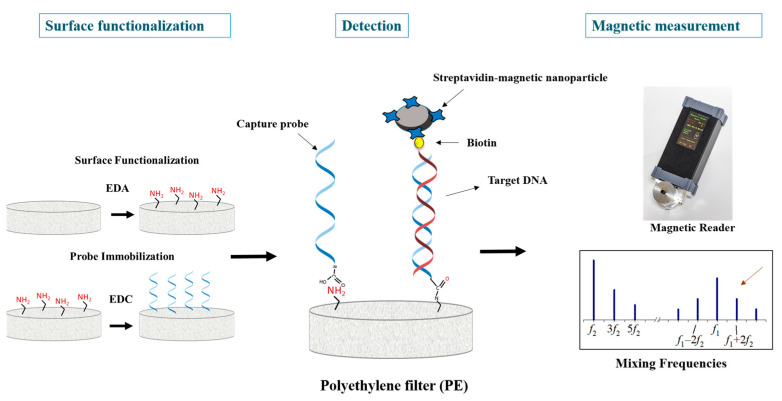
Magnetic nanoparticles-based DNA sensor for the detection of *Brucella* DNA. The red arrow marks the frequency mixing component that is demodulated.

The principle of the targeted detection of our designed magnetic nanoparticle-based DNA sensor is illustrated in Scheme 1. The developed assay is based on the magnetic sensing of the sequence-specific hybridization event involving the immobilized capture probe on an amine-modified PE filter and its complementary biotinylated target DNA. The strategy used entailed the selection of a DNA marker for *Brucella* bacteria based on screening and targeting sequence repeats that occur in multiple copies throughout the *Brucella* genome to obtain high sensitivity [23,24]. From the analyses of 38 repeats, we selected the target sequence, shown in Table S2. The target sequence is highly conserved (percent identity > 95%) among all *Brucella* representative genomes, with 5–6 copies distributed in the two chromosomes.

Only in the presence of the *Brucella* target sequence, the designed capture probes will bind specifically to the target sequence, achieving a hybridized structure. The hybridization event can be detected using magnetic nanoparticles as labels for magnetic sensing. Streptavidin-functionalized nanoparticles will bind to the target DNA by streptavidin–biotin interaction, thus allowing for the quantitation and detection of the DNA target analyte.

### 2.2. Surface Functionalization and Probe Immobilization

The selection of a proper immobilization strategy for coupling capture probes on a surface is vital for developing label-based nucleic acid assays [25]. The stability of the probe–surface bonds and the good orientation of the attached probe molecules are the main factors that impact the performance and efficiency of the hybridization assay. These requirements are achieved by the covalent immobilization method, which ensures a proper vertical orientation and a specific attachment of the probe and promotes the formation of a stable bond between the modified probe and the functionalized surface [26]. In our study, the well-known one-step EDC chemistry was used to covalently bind carboxylate-modified capture probes on amine-functionalized PE filters [27].

Polyethylene filters have no reactive functional groups on their surface; therefore, they need to be activated to generate oxygen and hydroxyl groups as reactive sites on the surface and then functionalized to introduce amine groups that enable the coupling with carboxylate-modified probes. A previous study showed that PE can be modified for the covalent immobilization of antibodies using ω-aminocellulose carbamate as a coating agent [28]. However, this modification requires the complex synthesis of ω-aminocellulose carbamate prior to its adsorption. In our study, we used a simple modification method consisting in incubating oxidized PE filters with an EDA solution for 1 h to generate amine groups on the filters’ surface.

To investigate the covalent binding of the probes, we performed a coupling reaction using the capture probes at 10 µM concentration, both on PE filters with EDC and on filters without EDC. Both target and nontarget ssDNA sequences at 5 µM concentration were tested for their hybridization ability, using filters without the capture probes as a control.

**Figure 1:**
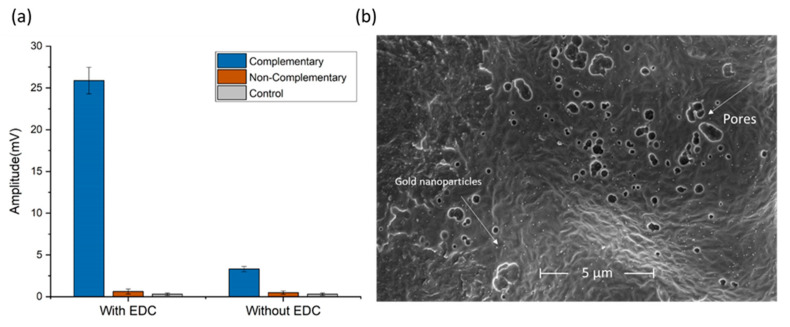
(**a**) The measured signal amplitudes of target and non-target (non-complementary) DNA at 5 µM concentration in the presence and absence of the EDC crosslinker. Error bars represent the standard deviation of three independent measurements. (**b**) SEM image of gold nanoparticles bound to the immobilized capture probes on a PE filter. Scale bar = 5 µm.

As shown in Figure 1a, in the presence of EDC, the signal amplitude of the target sequence was markedly higher than those of the nontarget sequence and the control. In the absence of EDC, the signal amplitude of the target sequence was noticeably slightly higher than those of the nontarget sequence and the control, which might be due to the electrostatic interaction between the negatively charged phosphate groups in the DNA and the positively charged amine groups on the functionalized surface. By comparing the signal amplitudes of the target and nontarget DNA in the presence or absence of EDC, we assumed that the coupling of the capture probe on the PE filter occurred through the specific EDC-dependent covalent coupling, and not by an electrostatic interaction. The results also indicated that the capture probe was selective toward the target sequences, as the average signal amplitudes of the nontarget DNA were very low.

To confirm the binding of the capture probes on the PE filters, the same EDC coupling strategy was employed on amine-functionalized and -non-functionalized PE filters using another capture probe of identical sequence and length but with biotin modification at the 3′ end. Streptavidin-conjugated gold nanoparticles (40 nm) were used as labels to enhance contrast for scanning electron microscope (SEM) visualization, as shown in Figures S1 and S2. Figure 1b shows the SEM image of the bound gold nanoparticles with the immobilized capture probes, confirming the binding of the probes on the functionalized PE filters.

### 2.3. Assay Optimization

To improve and maximize the signal amplitude of our DNA magnetic biosensor, we investigated and optimized several experimental conditions that would influence hybridization performance and magnetic sensing.

#### 2.3.1. Adjusting Probe Parameters

The amount and distribution of bound capture probes on a solid surface are key factors in achieving efficient hybridization between the immobilized probes and their complementary targets [29]. Thus, selecting an optimal probe concentration to ensure the best hybridization kinetics is needed to avoid unwanted behaviors that would affect the duplex formation, such as steric hindrance and electrostatic repulsion that occur at high probe concentration and signal reduction observable at low probe concentration [30].

**Figure 2:**
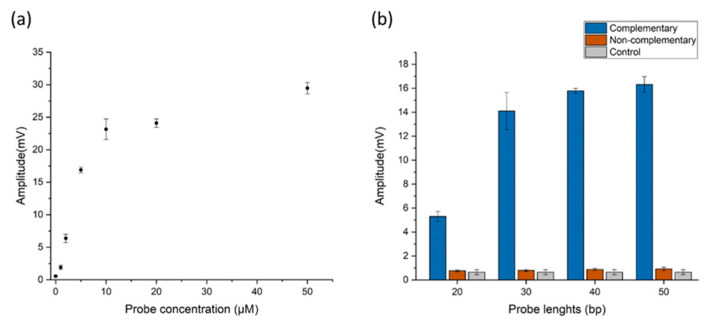
(**a**) Effect of probe concentration on signal amplitude. The concentrations of the capture probe were 50, 20, 10, 5, 3, and 1 µM. The target DNA concentration was 5 µM. (**b**) Effect of probe length on signal amplitude. The probe lengths were 20, 30, 40, and 50 bp. Error bars represent the standard deviation of three independent measurements.

Therefore, we optimized our assay using different concentrations of the capture probes, ranging from 1 µM to 50 µM. As shown in Figure 2a, increasing the concentration of the capture probes increased the signal amplitude. This clearly indicated that a high number of probe molecules bound on the PE filter increased the hybridization efficiency between the probes and their complementary DNA, thus increasing the signal amplitude. When a low concentration of probe (1 µM) was tested, the signal amplitude was low. When a very high concentration of probe (50 µM) was tested, the possible interference due to steric hindrance was not observed, and the signal amplitude obtained was the highest. This observation can be explained by the availability of a sufficient surface area on the PE filter to bind large amounts of capture probe. In fact, this explanation is supported by the SEM image in Figure 1b, which demonstrated relatively large distances between adjacent probes distributed on the PE filter.

For the selection of a suitable probe concentration for our assay, we considered that the assay would be used mainly in developing countries where a low cost is an essential factor. So, a low amount of probe concentration and a high signal-to-background ratio were considered as criteria for our selection. As a result, the 5 µM concentration was selected for further optimization experiments as a compromise, yielding the highest signal amplitude per probe concentration, as shown in Figure 2a.

We decided to investigate another factor that has a significant impact on the specificity and efficiency of DNA hybridization, which is the length of the immobilized capture probes. Several studies on DNA microarrays have shown that the signal strength is significantly affected when using different probe lengths [31,32,33]. The results of these studies confirmed that using longer probes provides higher detection sensitivity than using shorter probes. In principle, the longer the probe, the more accessible it is from a surface to its complementary DNA sequence, hence increasing the probability of hybridization and thus the detection sensitivity. In addition, a higher number of hydrogen bonds formed in duplex DNA will lead to more stable hybrids. However, long probes are more prone to cross-hybridization with non-specific DNA sequences than short probes [34]. Therefore, it is critical to find a trade-off between binding efficiency and binding specificity by testing several probe lengths.

For our magnetic DNA biosensor, four probes were designed in different lengths (20 bp, 30 bp, 40 bp, and 50 bp) to detect the conserved sequence with the lowest variations within the selected target sequence. Target and DNA nontarget sequences at 5 µM concentration were used for hybridization and magnetic sensing. Figure 2b shows the performance of the designed probes for signal amplitude. The signal amplitudes obtained were higher when capture probes with lengths of 50 bp, 40 bp, and 30 bp were used. However, a significant reduction of about 50% in signal amplitude was obtained when a probe with 20 bp length was used. Thus, we selected the length of 50 bp as a proper probe length for the following experiments.

#### 2.3.2. Optimization of DNA Hybridization

**Figure 3:**
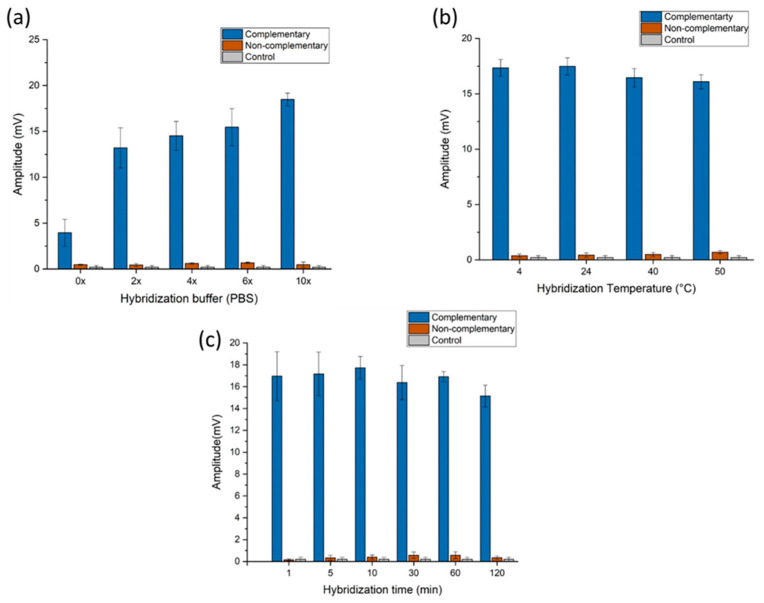
(**a**) Effect of ionic buffer strength. (**b**) Effect of temperature on signal amplitudes. (**c**) Detection time of the hybridization reaction. Capture probe and target and non-target DNA concentrations were 5 µM. Error bars represent the standard deviation of three independent measurements.

For the solid-phase hybridization assay, determining the optimal ionic composition is essential to ensure the best hybridization environment that will lead to a stable duplex formation. As DNA probes are negatively charged, the presence of cations is crucial to compensate the total negative charge, reducing the electrostatic repulsions that immobilized probes might exert [35]. In addition, a high-stability duplex would be ensured, as the association rate between probe and DNA increases at high salt concentration [36]. For our assay, several concentrations of the PBS hybridization buffer were prepared (10×, 6×, 4×, and 2×), and a distilled water solution was used as a control. A target and a nontarget sequence at 5 µM concentration were mixed with the prepared solutions and incubated for 1 h to allow the hybridization. Figure 3a shows the effect of the ionic strength of the PBS buffer on the signal amplitude. The result indicated that the signal amplitudes decreased with decreasing saline concentrations of the PBS buffer. By comparing the signal amplitudes of all solutions containing ions with that of the distilled-water solution (without ions), we could confirm that the presence of ions in the hybridization buffer is important to enhance the hybridization efficiency, as all solutions containing salt showed higher signal amplitudes. In our analysis, the highest signal amplitude was obtained using the 10× PBS buffer. This saline concentration of the hybridization buffer was thus selected as optimal for the following experiments.

The ability of the test to detect the desired target at different temperatures is crucial, especially when the goal is to develop a point-of-care (POC) testing device that is intended to be used in the field under different environmental temperatures. Therefore, we investigated the effect of temperature on our DNA sensor’s signal amplitude, using target and nontarget DNA sequences. The DNA molecules were incubated for detection at the hybridization temperatures of 4, 24, 40, and 50 °C for 1 h. As shown in Figure 3b, the signal amplitudes obtained were almost equivalent at all hybridization temperatures tested. This indicated that the performance of our developed sensor is consistent in a wide range of hybridization temperatures, which may minimize any possible influence of the external temperature both in cold and in hot regions.

To investigate the speed of detection and the influence of the hybridization time on signal amplitude, DNA target and nontarget sequences were incubated over several time periods. Some samples were added to the PE filters, and hybridization was carried out without incubation by gravity flow, at a flow rate ~200 µL/min. Other samples were first incubated for 5, 20, 30, 60, and 120 min. Figure 3c shows the influence of time on signal amplitude. It can be seen that the signal amplitudes obtained were high at all hybridization times and no significant differences were measured when the target DNA was used. However, the signal amplitudes were very low at all hybridization times when the nontarget DNA was used. The results showed that our proposed sensor worked rapidly and was capable of detecting the target DNA in 90 s of hybridization time.

#### 2.3.3. Magnetic Sensing Optimization

**Figure 4:**
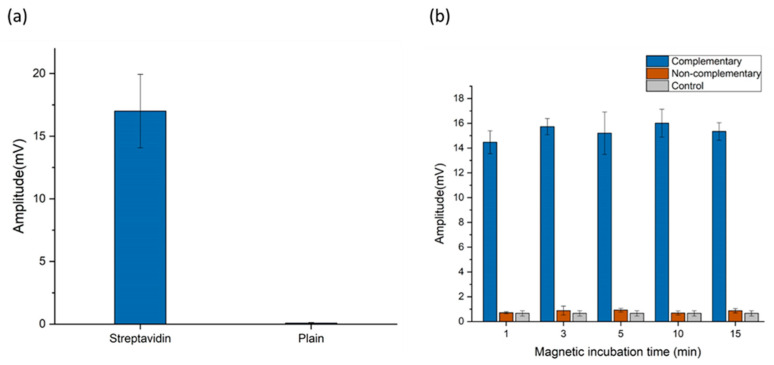
(**a**) The specificity of the binding of MNPs to biotinylated target DNA. (**b**) Effect of the MNP incubation time on signal amplitude. Error bars represent the standard deviation of three independent measurements.

To test the specificity of the binding of the magnetic nanoparticles to the biotinylated target DNA, another type of magnetic nanoparticles with the same size and specifications but no streptavidin shell (plain) were tested. Samples containing a DNA target were used at 5 µM concentration for hybridization and magnetic sensing. As seen in Figure 4a, the signal amplitudes of the magnetic nanoparticles with the streptavidin shell were substantially higher than those of the nanoparticles without the streptavidin shell. This result confirmed that the binding of the magnetic nanoparticles to the biotinylated target DNA was specific through biotin–streptavidin interactions.

To ensure that the maximum number of magnetic nanoparticles could bind to the biotinylated target DNA, the magnetic nanoparticles were added to PE filter samples containing target and nontarget sequences and tested at several incubation times (1, 3, 5, 10, and 15 min). The effect of the magnetic nanoparticle incubation time is shown in Figure 4b. High signal amplitudes were observed at all incubation times when the target DNA was used. In contrast, only a very low signal amplitude, at the same level as that of the control samples, was measured when the nontarget DNA was used. For our experiment, 3 min of magnetic nanoparticle incubation time was selected as the optimal time.

#### 2.3.4. Analytical Performance

**Figure 5:**
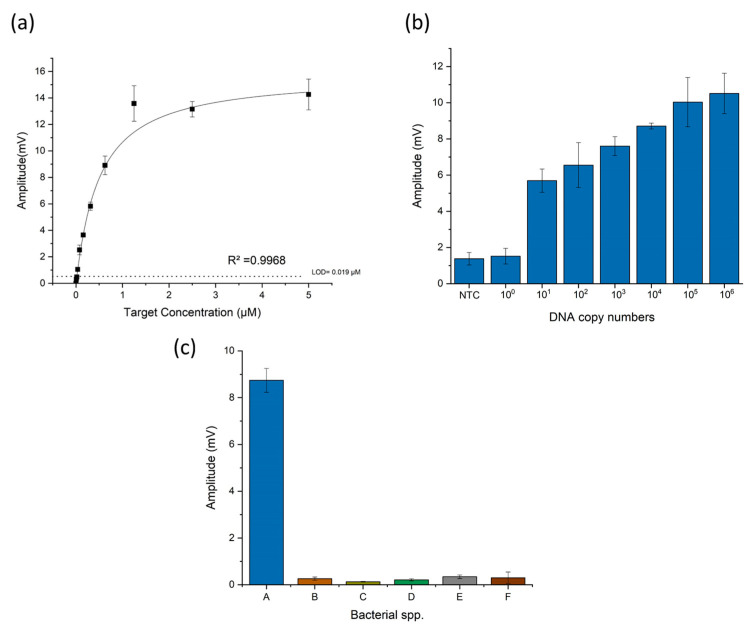
(**a**) The calibration curve of the measured signal amplitudes at different synthetic ssDNA concentrations. (**b**) The signal amplitudes for different copy numbers of amplified *Brucella* DNA. (**c**) The signal amplitudes for *Brucella melitensis* and non-related bacterial genomic DNA amplified by PCR. (A) *Brucella melitensis* at 2 ng/µL, (B) *Ovax Chlamydia* at 5 ng/µL, (C) *Campylobacter fetus* subsp. *venerealis* (*Cfv*) at 27 ng/µL, (D) *Campylobacter fetus* subsp. *fetus* (*Cff*) at 55 ng/µL, (E) *Escherichia coli* (*APEC*) at 83 ng/µL, (F) *Salmonella enteritidis* at 84 ng/µL. Error bars represent the standard deviation of three independent measurements.

The sensitivity and dynamic range of detection of our proposed DNA magnetic sensor were assessed under the previously determined optimized conditions. The selected capture probes with 50 bp size at 5 µM concentration were immobilized on PE filters for 30 min and then blocked with BSA for 1 h. After immobilization, serial dilutions of ssDNA targets were prepared ranging from 5 µM to 9.8 nM. For each concentration, triplicate samples containing 10 µL of the target were mixed with 290 µL of 10× PBS buffer saline. In addition, blank samples without the ssDNA targets were prepared to determine the limit of detection (LOD). The samples were added to the PE filters, and hybridization was carried out through gravity flow at a flow rate of 200 µL/min. After the hybridization, the magnetic nanoparticles were incubated for 3 min to allow their binding to biotinylated DNA. Finally, the samples were inserted into the measurement head of our portable magnetic reader to measure the signal amplitudes. A calibration curve was generated by fitting the mean and standard deviation of each ssDNA concentration after subtracting the background values using the Hill function presented in Equation (1). As shown in Figure 5a, the signal amplitudes increased with higher ssDNA target concentrations. The limit of detection was determined based on Equation (3) and was about 19 nM, with a linearity from 19 nM to 312.5 nM, a sensitivity of (22.1 + 1.2) mV/µM, and a detection range from 19 nM to 1650 nM. In addition, the coefficient of variation (CV) was calculated, and a value of less than 10% was obtained, showing the good reproducibility of the assay (Table S3).

To evaluate the capability of our assay to detect authentic *Brucella* genomic DNA, a serial dilution ranging from 5 to 5 × 10^6^ copies of the template was performed, followed by amplification using PCR and gel electrophoresis for visualization (Figure S3). The generated amplicons were then treated with lambda exonuclease to generate complementary ssDNA through the digestion of the undesired strand amplified by the phosphorylated reverse primers. Finally, the target ssDNA was added to 250 µL of 10× PBS buffer for hybridization and magnetic detection. As shown in Figure 5b, the sensor was able to detect the target at all concentrations ranging from 55 to 5 × 10^6^ copies, with high signal amplitude. However, the signal amplitudes were very low when five copies of the genome were tested, similar to those recorded for the non-template control (NTC). These results indicated that the assay has high sensitivity in detecting amplified *Brucella* DNA, being sensitive to amounts as low as 55 copies. Compared with other reported nanoparticles-based biosensors, the ability of our biosensor to detect very low concentration of *Brucella* DNA makes it highly suitable for the early and accurate diagnosis of brucellosis in field applications (Table 1). Moreover, the sensor specificity was also evaluated by analyzing the PCR-amplified products in the presence of gDNA from different types of bacteria, including *Campylobacter fetus* subsp. *venerealis* (*Cfv*), *Campylobacter fetus* subsp. *fetus* (*Cff*), *Ovax Chlamydia* vaccine, *Escherichia coli* (*APEC*), and *Salmonella enteritidis*. The selection of these bacterial species was based on their potential to interfere with *Brucella* detection, as they can coexist in animal samples subjected to examination. Notably, while the test showed a specific high signal when examining *Brucella melitensis* gDNA, all non-related bacterial genomes showed a very low signal amplitude, ensuring the specificity of the primers and capture probes toward *Brucella* DNA, as shown in Figure 5c and Figure S4. In this proof-of-concept experiment, we confirmed the capability of our sensor to detect genomic DNA. However, the methods used, like PCR and lambda exonuclease digestion, have limitations in regard to point-of-care testing. Therefore, we will focus on adopting alternative methods like isothermal amplification to establish a more practical DNA detection platform suitable for use in the field.

### 2.4. Potential for Future Development

The developed assay, which was validated as a proof of concept, is a promising point-of-care method with several interesting features for DNA detection. The assay can be easily applied to detect any type of microbial pathogen and is not limited to *Brucella*. It can also offer a fast and accurate solution in the fight against global pandemics. In addition, the inexpensive portable readout unit, the affordable magnetic nanoparticles, and the disposable polyethylene filters make it an attractive solution to perform large-scale field screening and ensure the accessibility of the technology to low-/middle-income countries. Moreover, the assay can be integrated with several isothermal amplification methods to achieve better performance and enhance its practicality in the field. However, further investigation will be crucial to establish its robustness and reliability and thus its possible contribution to global health care.

## 3. Materials and Methods

### 3.1. Target Selection and Capture Probes Design

The DNA repeats in the referenced genome of *Brucella melitensis bv. 1 str. 16M* were predicted using the in silico tool ‘Find Repeats’ on the Softberry website (http://www.softberry.com (accessed on 1 February 2022)). All repeats with more than 5 copies in the genome and that were more than 70 bp in length were selected for further screening. The conservation of the selected repeats at the *Brucella* genus level was confirmed by screening the selected sequences as queries against all completed representative genomes of *Brucella* spp. using the BLAST tool (https://blast.ncbi.nlm.nih.gov/Blast.cgi (accessed on 1 February 2022)). To determine the specificity of the candidate targets, the selected sequences were used as queries against all available genomic sequences in the nucleotide collection database, excluding the *Brucella* genus. Any sequence that did not fulfill the two conditions of (a) specificity to the *Brucella* genus and (b) conservation across all *Brucella* strains were excluded from further analysis.

For capture probe design, only the sequence with lowest variation within the selected target candidate was chosen for designing complementary capture probes. The capture probes were designed at four different lengths (50 bp, 40 bp, 30 bp, and 20 bp) to select the optimal size. A non-complementary DNA sequence (50 bp) was synthesized and used as a nontarget DNA to validate the binding specificity.

For the secondary structure analysis, the Unfold web server (DNA folding form) was used for predicting the secondary structures of all sequences [56]. The online software tool Oligoanalyzer (https://eu.idtdna.com/calc/analyzer (accessed on 20 February 2022)) was used to screen the formation of hairpin, self-dimer, and heterodimer structures.

### 3.2. PE Surface Modification

PE filters were placed inside a plasma oven for oxidation and treated for 5 min to activate their surface. The plasma conditions were t 50 sccm oxygen flux and 50 W power, as suggested by [57]. The plasma oven used was the oxygen plasma generator 100-E from PVA TePla Analytical Systems GmbH (Westhausen, Germany).

For surface amine functionalization, the activated filters were incubated in a diluted EDA solution at 1% concentration for 1 h with shaking at 15 rpm at room temperature; then, the PE filters were washed with distilled water and dried at room temperature.

### 3.3. Filter Preparation

To facilitate the diffusion of the solutions through the pores of the filters, the PE filters were inserted inside ABICAP columns and incubated in a 99% ethanol solution under negative pressure to remove air bubbles. The filters were then washed with distilled water and stored at room temperature for further use [20].

### 3.4. Capture Probe Immobilization

The coupling reagent EDC (10 mg) was added to a solution containing 10 µL of capture probes and 450 µL of MES buffer for the activation of the carboxyl groups. The solution was then added to the filters for the formation of covalent amide bonds between the activated carboxyl groups and the amine groups on the PE filter surface. The filters were closed with caps at the bottom and incubated at room temperature for the desired incubation times. The non-bound DNA probes were removed by washing the PE filters with 750 µL of 1× PBS by gravity flow. To avoid the nonspecific binding of the magnetic nanoparticles and the DNA targets to the filters, the PE filters were blocked with 500 µL of a 1% BSA blocker for 1 h.

### 3.5. Hybridization and Magnetic Labeling

A volume of 10 µL of complementary or noncomplementary 5′-biotinylated ssDNA sequences was mixed with 290 µL of PBS buffer. Each solution was added to the column filters for the hybridization reaction and incubated at room temperature for the desired time. The filters were then washed with 750 µL of 1× PBS buffer to remove the non-hybridized DNA sequences.

The magnetic nanoparticles were used as labels for sensing the hybridization between the probes and the target sequences. In this study, the magnetic nanoparticles were functionalized with a streptavidin shell which binds specifically to hybridized biotinylated sequences. For the labeling assay, 15 µL of the stock solution of magnetic beads (5 mg/mL) was added to 400 µL of 10× PBS and mixed by pipetting. The solution was added to the column filter and incubated for the desired time at room temperature. After incubation, a final washing step was performed using 750 µL of 1× PBS to remove the unbound magnetic nanoparticles.

### 3.6. Detection of Amplified Brucella Genomic DNA

Forward and reverse primers were designed to amplify the selected target sequence to obtain 84 bp target DNA that was complementary to the capture probes (Table S1). *Brucella* genomic DNA was amplified in 50 µL of PCR mixture containing 0.75 µM of both forward and reverse primers, 200 µM of dNTPs, 2 µL of DNA, 1× Phusion HF buffer, and 1 unit of Phusion DNA polymerase. The cycling conditions were as follows: initial denaturation at 95 °C for 10 min, followed by 30 cycles at 95 °C for 30 s, 60 °C for 20 s, and 72 °C for 5 s, and final extension at 72 °C for 10 min. The amplified DNA was then digested to generate ssDNA using 1 unit of lambda exonuclease at 37 °C for 15 min. After digestion, 250 µL of 10× PBS hybridization buffer was added to the amplicons for magnetic detection.

### 3.7. Frequency Mixing Magnetic Detection

The DNA target quantification and detection were achieved using the magnetic biosensing technique (FMMD) for superparamagnetic particle sensing [58]. In summary, the PE filter containing superparamagnetic nanoparticles was exposed to a time-varying magnetic field consisting of two distinct excitation frequencies, i.e., a low frequency (*f*_2_ = 63 Hz with 16 mT field amplitude) for driving the beads to magnetic saturation and a high frequency (*f*_1_ = 40.5 kHz with 1.3 mT amplitude) for probing the magnetization state. Due to their non-linear and non-hysteretic magnetization, the superparamagnetic particle response generated different new sum and difference mixing frequencies. Among these mixing frequencies, the frequency *f*_1_ *+* 2*f*_2_ showed the highest response signal and was therefore selected for sensing and quantifying the streptavidin-conjugated magnetic nanoparticles.

The sensing process was performed by inserting the column filters inside the measurement head of a small portable magnetic reader. The frequencies were generated by a cluster of coils implemented inside the measurement head for driving, excitation, and detection. All measurements were performed at room temperature.

### 3.8. Data Analysis

The measured signal amplitudes, including mean and standard deviation, were fitted to the nonlinear Hill function using software OriginPro 2019, V 9.6, Northampton, MA, USA, according to the equation

(1) $$y=end\frac{x^{n}}{k^{n}{\;+\;x}^{n}}$$

where *y* indicates the measured signal amplitudes, *end* is the highest concentration, *x* is the concentration of the ssDNA target, and *n* is the Hill coefficient.

(2) $$x={\left(\frac{y}{end-y}\right)}^{{}^{\frac{1}{n}}}$$

For quantification, the above inverted Hill function was used to determine and quantify the unknown concentration of ssDNA after subtracting the blank values from the measured signals.

For the limit of detection (*LOD*) determination, the following equation was used:

(3) $$LOD=Blank+3\cdot StDev\left(Blank\right)$$

## 4. Conclusions

In conclusion, we developed a new magnetic nanoparticle-based biosensor for POC detection and quantification of *Brucella* DNA. By utilizing the unique non-linear and non-hysteretic magnetization characteristics of superparamagnetic nanoparticles through frequency mixing magnetic detection technology, we successfully achieved a rapid, selective, and sensitive detection of *Brucella* DNA on a functionalized PE filter. The proposed magnetic biosensor, optimized and tested on synthetic and amplified gDNA, exhibited a high selectivity, robustness, and reproducibility, allowing for the reliable detection of *Brucella* DNA. A detection range from 19 nM to 1650 nM was found. When tested with products amplified from *Brucella* genomic DNA, the detection limit was as low as 55 copies.

Overall, the portability of the measurement device, the low cost of the reagents it requires, the fast detection time, and the ability to deliver quantitative results make this biosensor a valuable tool for early on-site diagnosis and monitoring of *Brucella* infections in resource-limited settings. Further studies and research should focus on selecting a proper isothermal amplification method that can be combined with our sensor to achieve even better performance.

## Figures and Tables

**Table 1:** Comparison of the properties of our magnetic biosensor with those of existing methods.

Method	Target Element	Assay Time	Safety	Stage of Infection	Cost	Sensitivity/LOD	Specificity	Ref.
**Blood culture**	Living bacteria	Days to weeks	Low	Early to Acute	Moderate to High	10–90%	100%	[10,37,38]
**Rose bengal**	Antibodies	Minutes	Moderate	Acute to Chronic	Low	75–100%	39–100%	[39,40,41,42,43]
**Standard agglutination**	Antibodies	Hours to days	Moderate	Acute to Chronic	Low to Moderate	75–96%	44–99%	[44,45,46,47]
**ELISA**	Antibodies	Hours	Moderate	Acute to Chronic	Moderate to High	98.7%	98.4%	[48]
**Quantum dot**	Antibodies	2 h	Moderate	Acute to Chronic	High	96.15%	94.12%	[49]
**Colorimetric**	Antibodies	Hour	Moderate	Acute to Chronic	Moderate	98.33%	100%	[50]
**PCR**	DNA	Hours	High	Early to Chronic	Moderate to High	93-100%	98–100%	[10,51,52]
**Colorimetric**	DNA	2 h	High	Early to chronic	Low to Moderate	3.32 pg/mL	100%	[53,54]
**Electrochemical**	DNA	1 h	High	Early to Chronic	Moderate	2.7 × 10^−20^ mol dm^−3^	100%	[55]
**Magnetic**	DNA	* Less than 1 h	High	Early to Chronic	Low to Moderate	0.09 fM	100%	This study

* The detection time was ≈10 min; the actual assay time depended on the amplification method used.

## Data Availability

The data presented in this study are available from the corresponding author upon reasonable request.

## Supplementary Materials

The following supporting information can be downloaded at https://www.mdpi.com/article/10.3390/ijms242417272/s1.
