# Supplementary material for: DNA Sensor for the Detection of Brucella spp. Based on Magnetic Nanoparticle Markers
Source: Int J Mol Sci. 2023 Dec 8;24(24):17272. doi: 10.3390/ijms242417272 (PMC10744106; doi:10.3390/ijms242417272)
Supplement: Supplementary file 1 [file ijms-24-17272-s001.zip › ijms-2745912-supplementary.pdf]

DNA Sensor for the Detection of *Brucella* spp. Based on Magnetic Nanoparticle Markers

Abdalhalim Abuawad [1,2], Yaqoub Ashhab [3], Andreas Offenhäusser [1,2] and Hans-Joachim Krause [1,*]

[1] Institute of Biological Information Processing: Bioelectronics (IBI-3), Forschungszentrum Jülich,
52428 Jülich, Germany; a.abuawad@fz-juelich.de (A.A.); a.offenhaeusser@fz-juelich.de (A.O.)

[2] Faculty of Mathematics, Computer Science and Natural Sciences, Rheinisch-Westfälische Technische Hochschule Aachen University, 52062 Aachen, Germany

[3] Palestine–Korea Biotechnology Center, Palestine Polytechnic University, Hebron P720, Palestine;
yashhab@ppu.edu

* Correspondence: h.-j.krause@fz-juelich.de

Contents

Reagents 2

Table S1. The designed target and capture probe sequences with their modification 3

Table S2. DNA target copy numbers and distribution in Brucella genome 4

Figure S1. SEM images of gold nanoparticles on amine-functionalized PE filter 5

Figure S2. SEM images of gold nanoparticles on non-functionalized PE filters. 6

Table S3. The coefficient values of Brucella DNA assay 7

Figure S3. Agarose gel images for the PCR of amplified Brucella DNA 7

Figure S4. Agarose gel images for the PCR of Brucella DNA and non-related bacterial genomes...........8

Supplementary Information

Reagents

1-Ethyl-3-(3-dimethylaminopropyl) carbodiimide (EDC), BupH MES-Buffered Saline Packs containing 0.1 M 2-(N-morpholino) ethanesulfonic acid, 0.9% sodium chloride at pH 4.7 (MES), phosphate-buffered saline (PBS) (10 mM phosphate, 150 mM sodium chloride, pH 7.3 to 7.5), and (10X) bovine serum albumin in PBS (BSA) were purchased from Thermo Fisher Scientific (Erlangen, Germany). Ethylenediamine (EDA) and gold nanoparticles with 40 nm size were obtained from Sigma-Aldrich Chemie GmbH (Taufkirchen, Germany). Phusion® High-Fidelity DNA Polymerase, and lambda Exonuclease kits were purchased from New England Biolabs GmbH (Frankfurt am Main, Germany). All solutions were prepared in ultrapure distilled water.

Sintered polyethylene filters (PE) with 2.5 mm X 5 mm size and about 20 µm pore size were obtained from Senova mbH (Weimar, Germany). The superparamagnetic nanoparticles (MNPs) used were both of type Synomag®-D, one has a streptavidin surface (Product code: 104-19-701) and the other has a plain surface (Product code: 104-00-701), and both having the same hydrodynamic diameter (70 nm). The MNPs were obtained from micromod Partikeltechnologie GmbH (Rostock, Germany).

Table S1. The designed oligo sequences with their modification were synthesized by biomers.net GmbH (Ulm, Germany). The letters "T", "C" and "P" refer to target, control and probe followed by a number which indicates the sequence length. The two letters "F" and "R" refer to forward and reverse.

**Oligonucleotide Name**	**Sequence (5′ to 3′)**	**Modification (5′)**	**Length (bp)**
**PCR Amplicon**	AGGCTGTAGTGGCGAATTAACTTGTGGTTTGGTATGGGCAAAAAATCTTCAG CTTTAGGAGCGAAACCGAAGGTGGAGTG GT TC		84
**T (50)**	GGTATGGGCAAAAAATCTTCAGCTTTAGGAGCGAAACCGAAGGTGGAGTG	Biotin	50
**C (50)**	TTCAAGTAGTCCAGGAGCCGTAAGGGATTGGACACCACGTGCAGTCACAG	Biotin	50
**P (50)**	CACTCCACCTTCGGTTTCGCTCCTAAAGCTGAAGATTTTTTGCCCATACC	COOH	50
**P (40)**	CACTCCACCTTCGGTTTCGCTCCTAAAGCTGAAGATTTTT	COOH	40
**P (30)**	CACTCCACCTTCGGTTTCGCTCCTAAAGCT	COOH	30
**P (20)**	CACTCCACCTTCGGTTTCGC	COOH	20
**F primer**	AGG CTG TAG TGA CGA ATT AAC TTG TGG	Biotin	27
**R primer**	GAA CCA CTC CAC CTT CGG TTT CGC TCC	Phosphate	27

Table S2. DNA target copy numbers and distribution in *Brucella* genome

Name	Accession numbers	Number of copies		Percent identity
		Chromosome 1	Chromosome 2	
**Brucella melitensis bv. 1 str. 16M**	NC_003317.1<br>NC_003318.1	5	1	(95- 100) %
**Brucella abortus 2308**	NC_007618.1<br>NC_007624.1	4	1	(95-98.8) %
**Brucella suis 1330**	NC_004310.3<br>NC_004311.2	5	1	(95-98) %
**Brucella ovis ATCC 25840**	NC_009505.1<br>NC_009504.1	5	1	(95-98) %
**Brucella canis ATCC 23365**	NC_010103.1<br>NC_010104.1	5	1	(95-98) %
**Brucella microti CCM 4915**	NC_013119.1<br>NC_013118.1	5	1	(95-98) %
**Brucella inopinata strain 141012304**	NZ_LT605585.1<br>NZ_LT605586.1	3	1	(91-96) %
**Brucella ceti TE10759-12**	NC_022905.1<br>NC_022906.1	5	1	95%

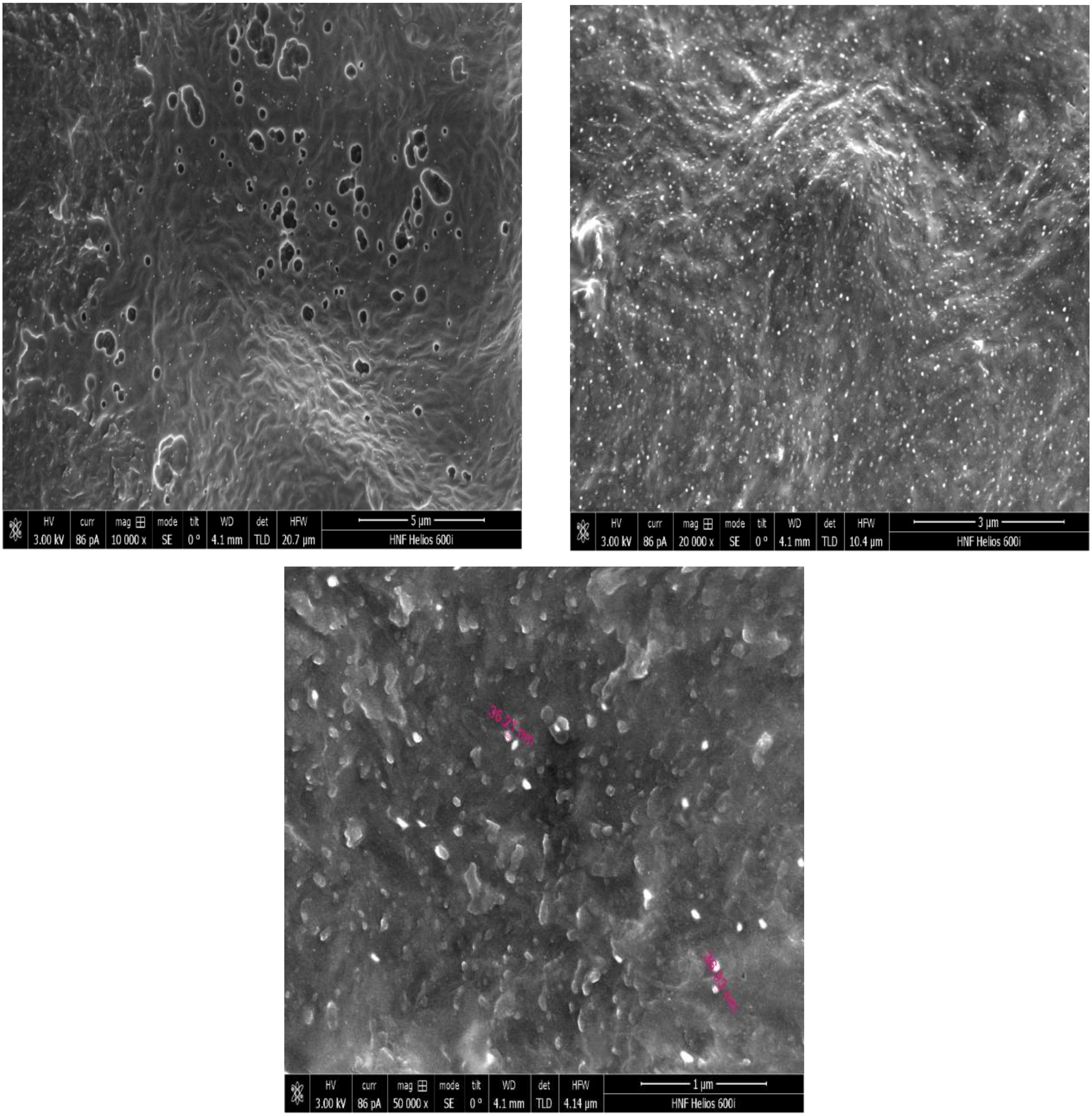

Figure S1. SEM images of gold nanoparticles on amine-functionalized PE filter.

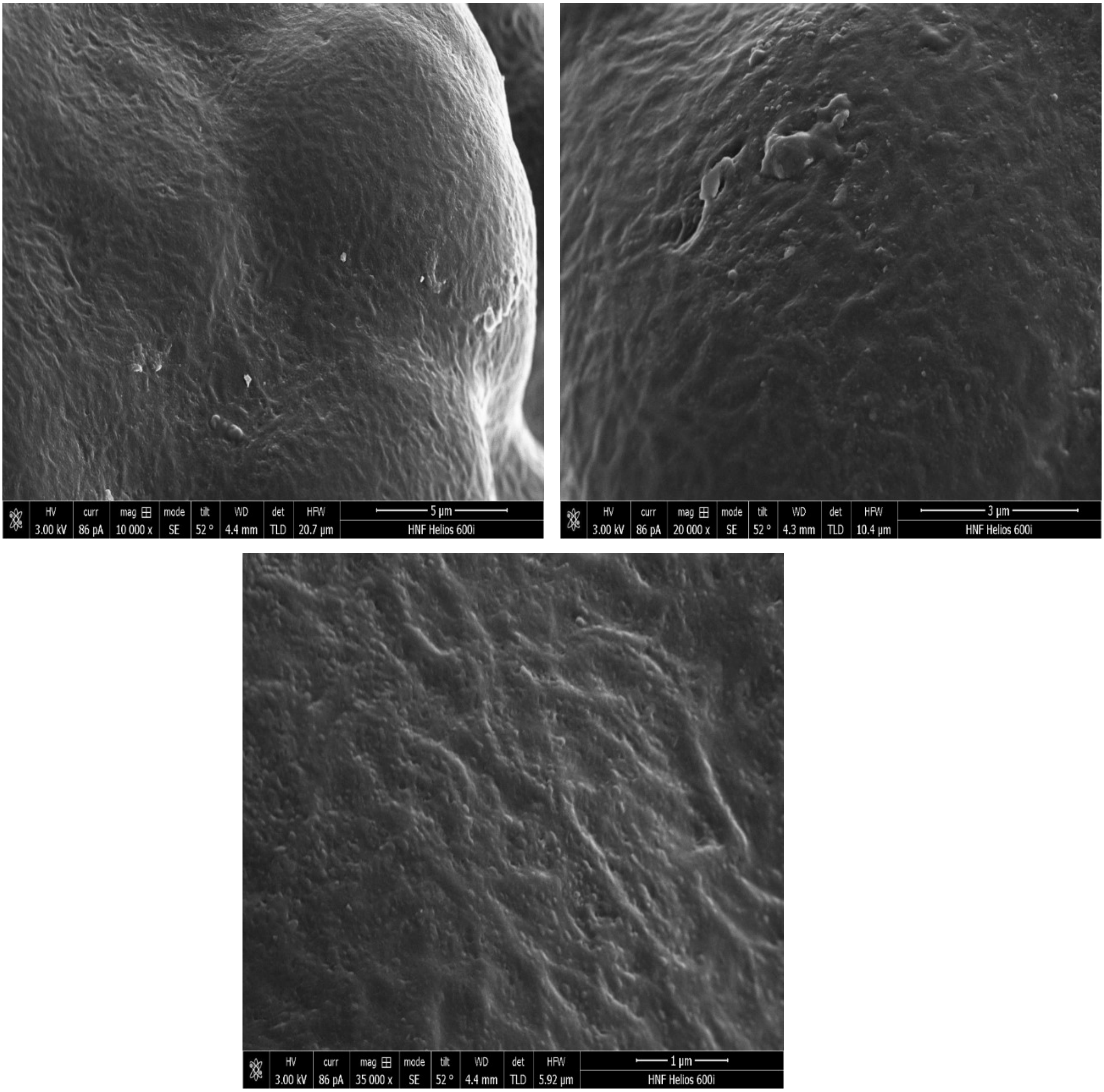

Figure S2. SEM images of gold nanoparticles on non-functionalized PE filters.

Table S3. The coefficient values of *Brucella* DNA assay.

ssDNA concentration	Intra-assay (n=4)			Intra-assay (n=4)		
	Mean (V)	S. D	CV	Mean (V)	S. D	CV
5 µM	0.01474	0.00121	8.22	0.01494	0.00105	7.008
1 µM	0.0099	0.00092	9.29	0.0103	0.00074	7.184
0.5 µM	0.00847	0.00065	7.6	0.0088	0.00063	7.15

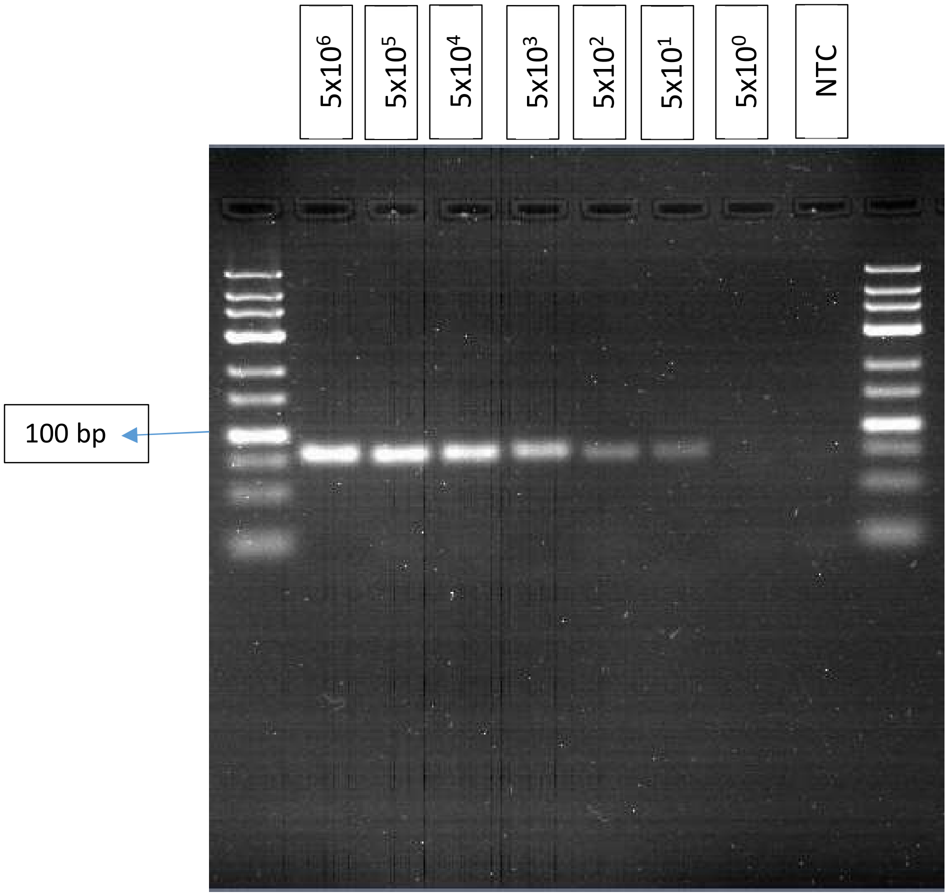

Figure S3. Agarose gel images for the PCR of amplified Brucella DNA (84 bp) with different copy numbers.

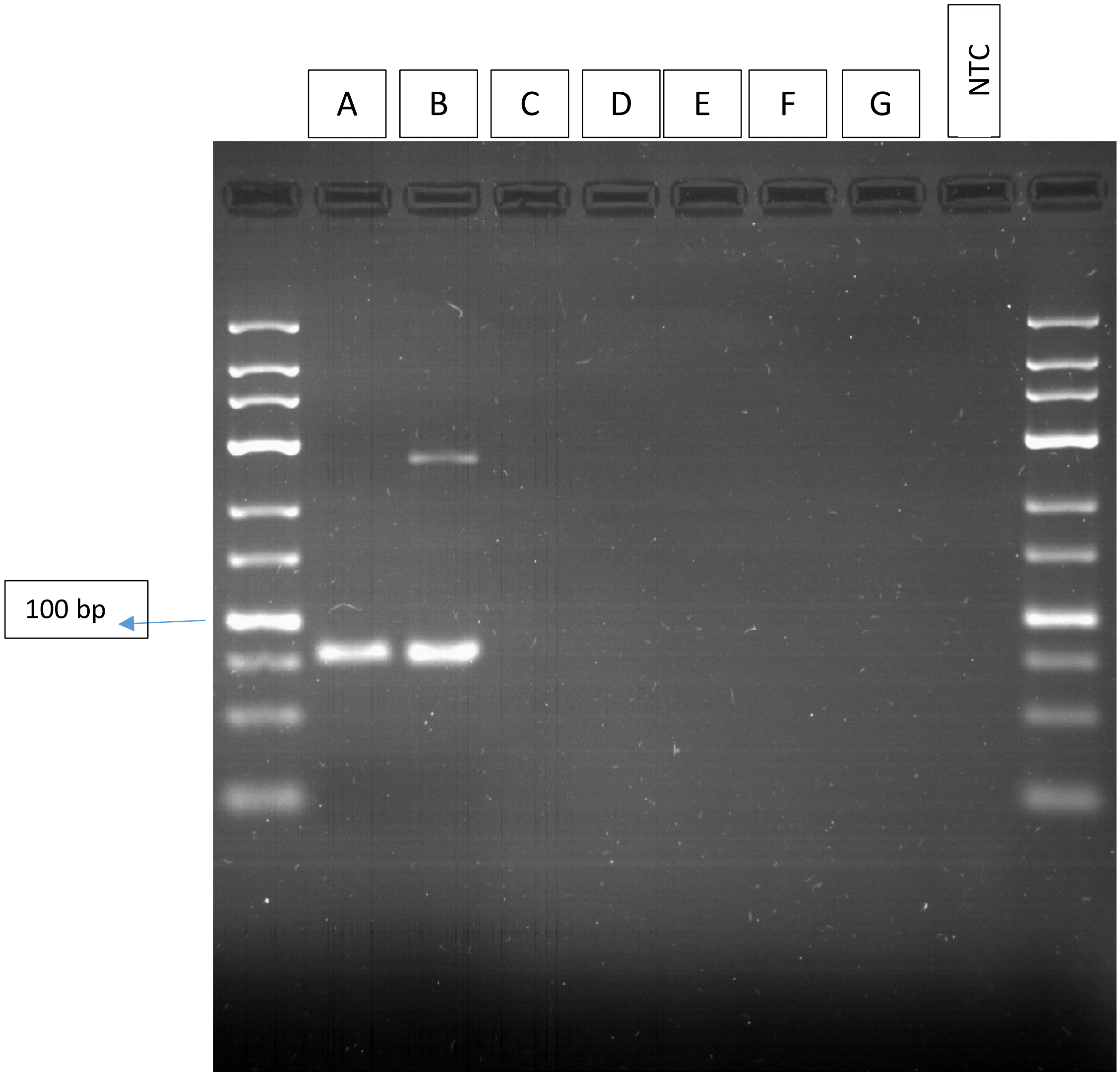

Figure S4. Agarose gel images for the PCR of Brucella DNA and non-related bacterial genomes. (A) Brucella positive control, (B) Brucella melitensis at 2 ng/µL, (C) Ovax Chlamydia at 5 ng/µL, (D) Campylobacter fetus subsp. ve-nerealis (Cfv) at 27 ng/µL, (E) Campylobacter fetus subsp. fetus (Cff) at 55 ng/µL, (F) Escherichia coli (APEC) at 83 ng/µL, (G) Salmonella enteritidis at 84 ng/µL
